# Evaluation of a Pilot Medication-Assisted Therapy Program in Kazakhstan: Successes, Challenges, and Opportunities for Scaleup

**DOI:** 10.1155/2012/308793

**Published:** 2012-12-10

**Authors:** Azizbek A. Boltaev, Anna P. Deryabina, Almas Kusainov, Andrea A. Howard

**Affiliations:** ^1^ICAP, Mailman School of Public Health, Columbia University, New York, NY, USA; ^2^Republican Applied Research Center for Medicosocial Problems of Drug Addiction, Pavlodar, Kazakhstan

## Abstract

*Study Aims*. Evaluate the quality and effectiveness of the medication-assisted therapy (MAT) pilot in Kazakhstan and review implementation context and related challenges. *Methods*. We performed a desk review of MAT policy and program documents and reviewed medical records at three MAT sites in Kazakhstan. MAT patients (*n* = 93) were interviewed to assess their perceptions of the program and its impact on their health, criminal, drug use, and HIV risk related behaviors as well as expenditures on nonprescribed psychoactive drugs. Persons injecting drugs who are not in treatment, MAT program staff, and other stakeholders were interviewed to obtain their perspectives on MAT. *Results*. Legislation supports introducing MAT as a standard of care for treatment of opioid dependence; however, its progress has been hampered by active opposition. Inadequate access and coverage, insufficient supply management, scarce infrastructure of narcological facilities, limited opportunities for staff development, and restrictive methadone dispensing policies compromise the quality of the intervention and limit its potential benefits. There were significant reductions in criminal, drug use, and HIV risk related behaviors in patients receiving MAT. *Conclusions*. The MAT pilot in Kazakhstan demonstrated its feasibility and effectiveness in the local context and is recommended for scaleup throughout the country.

## 1. Introduction

Kazakhstan faces a concentrated HIV epidemic, with drug use being the most important risk factor for HIV transmission [1, 2]. The country is located on a major drug trafficking route from Afghanistan, resulting in the availability of inexpensive heroin and a high prevalence of drug use in the country, an environment that is increasingly conducive to the spread of HIV and other blood-borne infections. According to the national HIV integrated biobehavioral surveillance data from 2010, the estimated number of people who injected drugs (PWID) during the last 12 months was 119,140, which is 3.5-times higher than the number of PWID officially registered with the drug addiction treatment service.

Medication-assisted therapy (MAT), more widely known in the region as opioid substitution therapy (OST), is a rigorously evaluated and evidence-based medical intervention to treat opioid dependence that consists of prescription of methadone or buprenorphine as a replacement for illicit street opioid narcotics such as heroin. Research conducted to date has generated a great amount of evidence demonstrating that MAT in combination with psychosocial support produces the best outcomes in terms of reduced frequency of illicit drug use and injections, decreased criminal behavior, and improved social functioning [3]. 

MAT was initiated in Kazakhstan in October 2008 as a pilot intervention within the national multicomponent HIV project funded by the Global Fund to Fight AIDS, Tuberculosis, and Malaria (GFATM). By March 2012, MAT was implemented at three sites (Pavlodar, Temirtau, and Ust-Kamenogorsk), with a cumulative enrollment of 265 patients, of whom 118 were still actively enrolled in the program. 

Between February and March 2012, ICAP-Columbia University designed and implemented an assessment to determine the strengths and challenges of the MAT program in Kazakhstan at the request of the Ministry of Health (MOH). The purpose of this assessment was to determine the extent to which the program complies with the minimal recommendations developed by the World Health Organization (WHO) for psychosocially assisted pharmacological treatment of opioid dependence [3]. Specific objectives included to (1) describe the existing models of providing MAT to PWID; (2) assess the quality and efficacy of current MAT models; and (3) provide specific and feasible recommendations to the MOH based on identified capacity building needs to improve the quality, efficiency, and effectiveness of MAT. 

## 2. Materials and Methods

The assessment was conducted using qualitative and quantitative research methodologies including (1) systematic review of relevant documents; (2) semistructured interviews with key stakeholders and staff involved in the provision of MAT at narcological ^1^ centers (*n* = 18); (3) semi-structured interviews with opiate-dependent persons not in treatment (*n* = 32), as well as patients who were enrolled in MAT for more than three months (*n* = 93); (4) MAT site/narcological facility assessments (*n* = 3); (5) and medical chart review (*n* = 227). Information was triangulated across the various data collection methodologies to assess the outcome measures described below.

 For individual interviews with MAT patients, we used a standardized structured questionnaire that included validated research instruments including the Treatment Perceptions Questionnaire (TPQ) [4], the Drug use and Criminality section of the Opiate Treatment Index (OTI) [5], and the HIV Risk Questionnaire-Short Version [6]. In addition, questions related to patients' overall satisfaction with their own health and their drug-related expenses in the last 30 days were included. Similar variables related to participants' status one month prior to their admission to MAT were collected using the timeline follow-back (TLFB) methodology. TLFB is a measurement tool developed to help respondents to recall their behaviors, including substance use and sexual activities, through construction of an event calendar that provides memory cues for recollection of required details of the past [7, 8]. Each TPQ item was scored on a five-point scale, where 0 means strongly disagree and 4 means strongly agree. Scores for negative items were recoded to measure positive evaluations on all measures, with higher scores indicating greater satisfaction [9]. 

To evaluate the patients' satisfaction with existing services, the semi-structured portion of the questionnaire included items related to accessibility of drug dependency treatment services, personal experiences with using narcological services, perceived quality of services, cost considerations, potential and actual reasons that could lead to drop out from treatment, factors that could improve uptake and adherence to MAT, unmet needs related to drug dependence and HIV-related treatment services, and perspectives on improvement of services. Demographics and clinical characteristics of MAT patients who participated in interviews are shown in Table 1. 

 Interviews with PWID were conducted at all three MAT sites. Interviewers collected information about respondents' knowledge and attitudes about MAT, the accessibility of and barriers to narcological care and HIV prevention services for PWID, as well as potential ways to improve the current situation.

Medical charts and registers were reviewed by the assessment teams to assess the scope and quality of existing MAT services. A standardized data abstraction form was used to measure the nine indicators indicated in Table 3.

### 2.1. Statistical Analysis

Means and standard deviations were calculated separately for each TPQ item. Paired samples *t*-tests were used to compare differences in mean OTI scores and mean drug-related expenses 30 days prior to MAT enrollment and during the last 30 days on MAT. The Wilcoxon Signed Ranks test was used to compare the patients' satisfaction with their health and criminal behavior before and during MAT. Associations between MAT enrollment and HIV risk behaviors were evaluated with McNemar's test. A *P* value <0.05 was used as the threshold for significance in all analyses.

## 3. Results

### 3.1. Sociopolitical Environment

The sociopolitical environment around MAT in Kazakhstan is ambiguous. Kazakhstan leadership demonstrates strong support for MAT: in 2005, President Nazarbayev urged Kazakh healthcare to introduce innovative methods of HIV prevention, including the use of methadone to treat drug users [10, 11], and MAT was included in the National Healthcare Program “Salamatty Kazakhstan” and budget [12] as a measure to prevent HIV among PWID. However MAT is actively opposed in mass media publications and public campaigns by different groups, including medical specialists and community organizations. As a result of this active opposition [13], MAT implementation has been limited to only three sites with a total ceiling of 150 opioid dependent persons allowed for enrollment.

 Implementation of the existing MAT program is regulated by the MOH order on expanded access to MAT in Kazakhstan [14] and is implemented in accordance with clinical guidelines elaborated by the Republican Applied Research Center on Medical and Social Problems of Drug Abuse (RARC) [15], which are largely based on the WHO guidelines on psychosocially assisted pharmacological treatment of opioid dependence [3]. The guidelines include 18 years of age or older as one of the eligibility criteria; however available evidence does not suggest any contraindications to including younger age groups in MAT, nor does it demonstrate that nonopioid maintenance types of treatments are more effective in younger age groups [16]. Two other inclusion criteria that cause concern include the requirement for at least a three-year verified history of injecting drugs and/or at least two documented unsuccessful treatment attempts. Although excluding “fresh” opioid users who have not yet developed dependence is reasonable, patients' ability to prove the duration of their drug use and previous treatment attempts may be limited, especially for people who use anonymous treatment services to avoid inclusion in the official register of drug dependent individuals. Another obstacle that discourages PWID from entering MAT programs is the limited number of sites in the country and regulations forbidding take-home doses which prevent patients from travelling far from the treatment site without stopping MAT.

### 3.2. Costing and Financing

Currently MAT is virtually free for patients in Kazakhstan and the existing MAT sites are fully supported by the GFATM. Over 1.2 billion Kazakh tenges (approximately 8.5 million US dollars) were allocated for MAT by the MOH from 2011 to 2015; however these funds are not being spent because of the logistical difficulties related to local procurement of methadone described below. 

### 3.3. Procurement and Supply Management of Commodities

Methadone and buprenorphine are included in the list of medical substances that are strictly controlled by national regulatory bodies and their import, storage, and administration require special permissions in accordance with the law on narcotics, psychotropic substances, precursors and counteracting measures to prevent illegal circulation and abuse of these substances [17]. In part due to the limited market capacity, neither methadone nor buprenorphine are officially registered in Kazakhstan. This significantly complicates the import of methadone and makes it virtually impossible for local MAT providers to procure it independently. 

Currently methadone is procured by the Republican AIDS Center (RAC), the primary recipient of the GFATM HIV grant. Procurement is done based on the forecast provided by the RARC that collects information on methadone stock and potential demand from each MAT site on a monthly basis. Results of key stakeholder interviews show that this procurement mechanism is not very effective and the complete procurement cycle (from forecasting and planning to product delivery) takes as long as six to nine months. Current constraints in procurement management resulted in stockouts in 2010, when sites had to significantly reduce daily doses of methadone provided to patients during two months. Methadone supply interruptions lead to the loss of patients who could not continue to participate in the program with insufficient doses; some of these patients went back to using heroin. 

### 3.4. Human Resources

All three MAT sites have standard staffing structures that include a site coordinator, two narcologists, two nurses, a pharmacist, a social worker, and a psychologist. All MAT staff members were recruited from the same narcology clinics and MAT-related functions are performed in addition to their main jobs. The percentage of staff time devoted to MAT varies from 25% to 50% depending on the number of the patients, their needs, and the number of scheduled procedures such as individual and group therapies, and toxicology tests.

The majority of narcologists participating in the pilot project learned how to use methadone during various trainings and study tours organized by international development partners within and outside of the country. None of the nurses working in MAT sites received any formal training on MAT.

RARC has a three-day training module that was developed based on the existing MAT guidelines and various international training materials and is mandatory for all MAT clinicians. However, none of the medical schools include opioid substitution therapy as a part of their curricula on drug dependency treatment, which may contribute to the biased attitude towards MAT among medical professionals. 

### 3.5. Current Models of MAT and Their Infrastructure

All three MAT sites are located in the premises of local narcological dispensaries. In Pavlodar and Ust-Kamenogorsk MAT sites are located in areas with good access to public transportation while in Temirtau patients experience difficulties accessing the site due to limited public transport in that area. Methadone dispensing rooms in Pavlodar and Ust-Kamenogorsk are located among several other rooms where narcologists see ambulatory patients. In Temirtau, next to the MAT dispensing room there is a medical correction department, also known as a drunk tank that provides sobering up services for intoxicated people brought in by police. The proximity of these services demotivates some patients from going to the MAT site on a daily basis due to their fear of coming into contact with police or other persons they might know.

MAT sites in Pavlodar and Ust-Kamenogorsk operate from 8:00 am to 10:00 am and from 5:00 pm to 6:00 pm seven days a week, while in Temirtau MAT site operations start at 10:00 am, which makes it difficult for patients to receive methadone before work. Only MAT sites in Pavlodar and Ust-Kamenogorsk have separate rooms where clinical staff can provide counseling to patients in a confidential environment.

In Pavlodar, the MAT dispensing space is adjacent to a needle exchange point administered by the Oblast AIDS Center where PWID can receive HIV counseling, exchange syringes, and needles and obtain condoms for free. In the same end of the corridor there is a room provided by Pavlodar Narcology center to a nongovernmental organization, “INSIDE,” where MAT patients socialize and organize self-help groups and seminars with health specialists, including the Center's psychologist. Pavlodar has also arranged for an HIV specialist from the Oblast AIDS Center to work part time at the MAT site, to offer integrated HIV care to MAT patients. 

### 3.6. Monitoring and Evaluation

Variables collected and reported by each MAT site to RARC and RAC are shown in Table 2. Although semistandardized paper-based patient records are completed for all MAT patients, MAT sites have limited capacities for proper collection and documentation of data reported to RARC. As such, MAT sites differ in the ability to screen for viral hepatitis and provide other laboratory tests (this is discussed in more detail later in the paper); psychological assessments are conducted inconsistently and in some instances such assessments are conducted using nonstandard tools with unknown reliability. There are inconsistencies among MAT sites on the forms used for recording toxicology test results and the content of counseling sessions. The use of paper-based medical records makes it difficult to conduct data quality assessments and track overall treatment results. In 2011 uncertainty about the continuation of the MAT program led to a yearlong disruption in data collection and reporting to RARC.

### 3.7. Results of Medical Chart Review

Results of the medical chart review are summarized in Table 3. According to the existing MAT guidelines in Kazakhstan, the spectrum of services provided to MAT patients should include MAT, diagnosis of viral hepatitis, HIV and other sexually transmitted infections, and psychosocial care. Attending physicians are required to elaborate individual treatment plans addressing medical complications for each patient [15]. All MAT patients are tested for HIV on an opt-out basis; however, only one of the three MAT sites provides treatment of nonnarcological illnesses and is actively engaged in ART counseling and monitoring.

Chart review revealed that documentation of complete clinical reviews is not practiced and is not required by the existing MAT guidelines. However, clinical staff at all three MAT sites indicated that all patients undergo review by all specialists (narcologist, psychologist, and social worker) and urine toxicology tests are performed on a quarterly basis and that the results of these reviews are communicated within the MAT team and to the patient.

The percentage of patients screened for both HBV and HCV ranged from 55% to 85%. The low rate of HBV and HCV testing was mainly due to the fact that only one site (Temirtau) performed HBV and HCV testing on site. In the other two sites, HIV-positive patients are referred for free HBV and HCV testing to the local AIDS Centers and HIV-negative patients to private laboratories where services are expensive and often unaffordable for patients. 

The percentage of MAT patients with at least one counseling session during the last month differed significantly between sites. This is mainly due to the fact that in Pavlodar many patients attended group psychotherapy which was not systematically recorded in patients' charts; in Temirtau specialists do not have an ability to conduct individual counseling sessions with their patients due to a shortage of rooms and thus only organize group-counseling sessions that also are not recorded in individual patient records. 

The vast majority (95%) of MAT patients in Pavlodar and Ust-Kamenogorsk remained free from nonprescribed opioids six months after initiation of MAT, while in Temirtau over two-thirds of patients (68%) remained free from non-prescribed opioids at six months. Temirtau's comparably lower percentage of patients who remained opioid free is in part due to the fact that 22% (*N* = 5) of patient charts did not have urine toxicology tests results for the indicated time interval and thus were counted as positive for opioids.

In all sites more than a half of all enrolled patients remained in care for at least six months, with retention rates ranging from 55% to 72%. Reasons for discontinuation of MAT are summarized in Table 4. The highest percentage of patients discharged from the MAT program due to the continued breach of MAT program rules including regular omitted methadone doses (32%) were registered in Temirtau. This is in part explained by the late opening hours, inconvenient location, and inability to obtain individual counseling at the site. It is also important to note that among all patients who prematurely discontinued MAT, 22% did so in order to undergo inpatient treatment.

The majority of MAT patients in Pavlodar (92%) and Ust-Kamenogorsk (84%) remained free from non-prescribed opioids at twelve months after the initiation of MAT. Temirtau's comparably lower percentage of patients who remained free from opioids at twelve months after initiation of MAT is in part due to the fact that 50% (*N* = 11) of patient charts did not have urine toxicology test results recorded for the indicated time interval. According to MAT staff, these patients refused to undergo periodic toxicology tests. This situation, besides highlighting a potential source of unidentified positive test results, indicates gaps in the staff's capacity to motivate patients to follow project rules.

The proportion of patients on MAT remaining in care twelve months after the initiation of MAT ranged from 46% to 61% (Table 3). Of note, Temirtau had the highest proportion of dropouts from MAT by patients that needed to undergo treatment in various inpatient clinics. It is reasonable to assume that if it were possible to continue MAT in such clinics, a significant number of these patients would have remained in MAT, and thus the retention rate in Temirtau could have been comparable with the same indicator at the other two sites. 

International evidence suggests that the optimal daily dose of methadone is between 60 and 120 mg [3], although higher doses of methadone are associated with better clinical outcomes [18]. According to patients interviewed at MAT sites, many try to avoid increasing their methadone dose due to fears of disruptions in supply or discontinuation of the pilot MAT project. However, all clinicians interviewed report practicing flexible methadone dosing depending on patients' individual needs and health conditions. 

### 3.8. Patients' Satisfaction with the Program and Their Own Health Status

Patients' overall satisfaction with MAT was average to low: the highest mean score of 2,96 (SD = 0,47) was in Pavlodar, followed by Ust-Kamenogorsk and Temirtau where patients' overall satisfaction was scored 2,63 (SD = 0,37) and 2,40 (SD = 0,42), respectively. In Temirtau and Ust-Kamenogorsk MAT patients gave considerably low scores to how well they have been informed about decisions made regarding their treatment: 0,95 (SD = 0,38) and 0,72 (SD = 0,45), respectively; however patients in Pavlodar rated their satisfaction highly in the same domain (M = 3,29;  SD = 0,46). 

**Figure 1:**
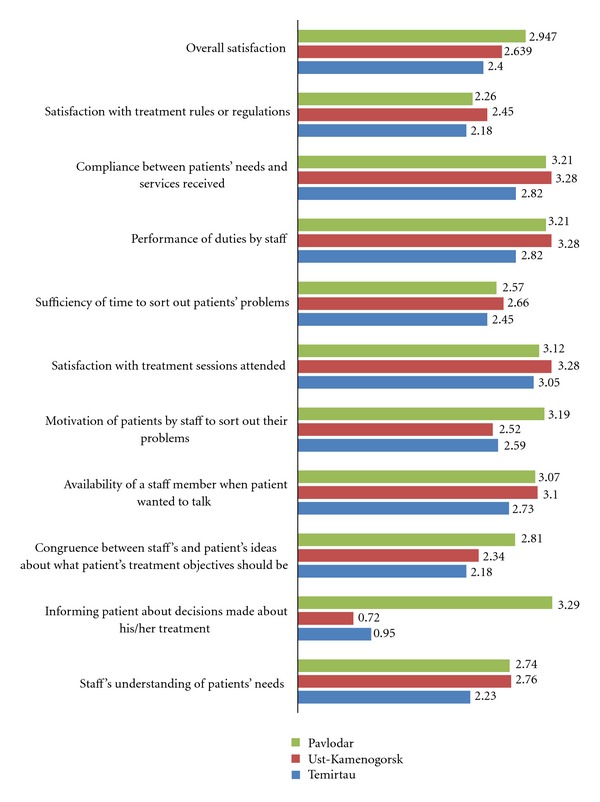
Clients' satisfaction with medication-assisted treatment services in Kazakhstan as indicated by responses to the Treatment Perception Questionnaire. Mean scores are depicted, where 0 means strong dissatisfaction and 4 means strong satisfaction.

Patients' level of satisfaction with their dose of methadone was the highest compared to the global mean score calculated on TPQ: in Pavlodar, Ust-Kamenogorsk, and Temirtau patients scored 3,36 (SD = 0,53), 3,48 (SD = 0,51) and 3,14 (SD = 0,47), respectively, on the item assessing the adequacy of the methadone dose they received to avoid experiencing withdrawal symptoms and craving drugs. Results of the treatment perception questionnaire are provided in Figure 1.

**Figure 2:**
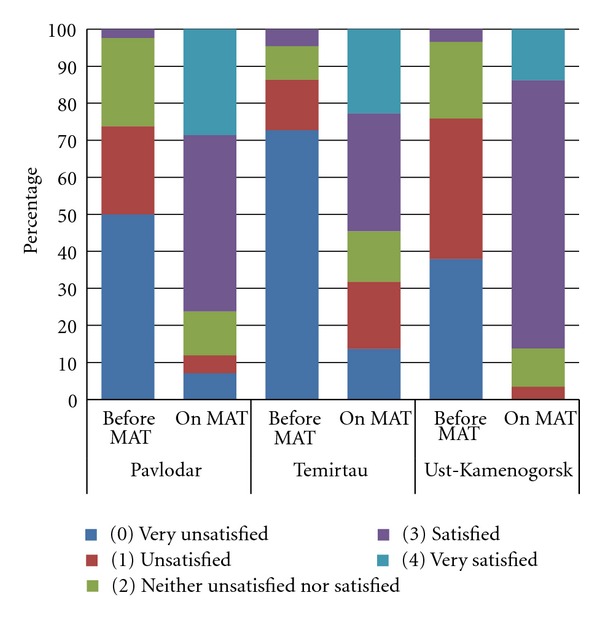
Level of satisfaction with one's own health status in the last 30 days before and after enrollment in medication-assisted therapy, by site.

MAT patients were asked “How satisfied with your health status were you during the last 30 days (and before starting MAT)?” and requested to choose one answer on a scale ranging from 0 (very unsatisfied) to 4 (very satisfied). As shown in Figure 2, there were statistically significant improvements in patients' perception of their health status compared to the period before initiating MAT in Pavlodar (median (before MAT) = 0.50, median (on MAT) = 3.0, *Z* = −5.337,  *P* < 0.001); Temirtau (median (before MAT) = 0.00, median (on MAT) = 3.00, *Z* = −3.486,  *P* < 0.001); Ust-Kamenogorsk (median (before MAT) = 1.00, median (on MAT) = 3.00, *Z* = −4.662,  *P* < 0.001).

 Our observation of a relatively low level of perception of MAT by patients and a significant increase in their health satisfaction after enrollment to MAT suggests that many of them did like the methadone but did not like the way in which services were delivered.

### 3.9. Evaluation of Patient Behaviors (Sexual, Drug Use, and Criminal)

The Opioid Treatment Index (OTI) was used to assess frequency of the use of psychotropic drugs for nonmedical purposes by patients during the last 30 days before their enrollment in MAT and the last 30 days prior to the interview.  Table 5 shows how the results were interpreted.

#### 3.9.1. Heroin

**Figure 3:**
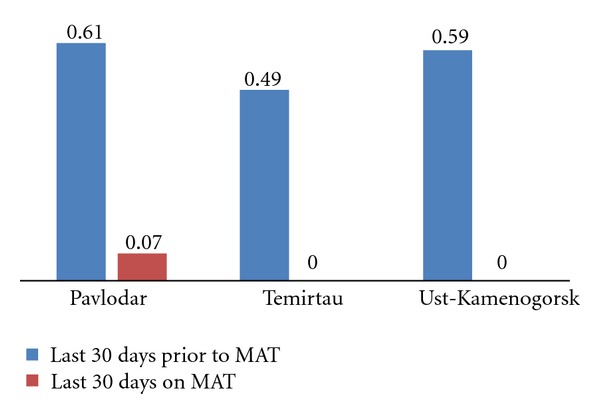
Use of heroin according to mean Opiate Treatment Index scores in patients before and after enrollment into medication-assisted treatment, by site.

 Paired *t*-tests demonstrated a significant difference in the frequency of heroin use by patients during the last 30 days prior to starting MAT and during the last 30 days on MAT across all three sites: in Pavlodar patients' heroin use frequency index dropped from 0.61 prior to MAT (SD = 0.67) to 0.07 (SD = 0.46);  *t*(41) = 4.09,  *P* < 0.001), while in both Temirtau and Ust-Kamenogorsk the indexes of heroin use frequency were reduced from 0.49 and 0.59, respectively, to 0.00, or in other words respondents reported that they did not consume any heroin during the last 30 days on MAT (In Temirtau, (M = 0.49,  SD = 0.44) and (M = 0.00,  SD = 0.00);  *t*(21) = 5.28,  *P* < 0.001), and in Ust-Kamenogorsk (M = 0.59,  SD = 0.76) and (M = 0.00,  SD = 0.00);  *t*(28) = 4.2,  *P* < 0.001).  Figure 3 demonstrates mean differences in heroin use. The relatively low level of heroin use prior to MAT enrollment can be explained by the fact that many MAT clients were enrolled at a time when they did not have easy access to heroin, which stimulated them to enroll in the program. In fact, a number of patients interviewed indicated that they were in opioid withdrawal when they enrolled in MAT. Over 90% of active PWID that participated in interviews reported daily use of heroin which may better reflect MAT patients' “normal” drug use patterns prior to facing difficulties in sourcing heroin.

#### 3.9.2. Opiates

**Figure 4:**
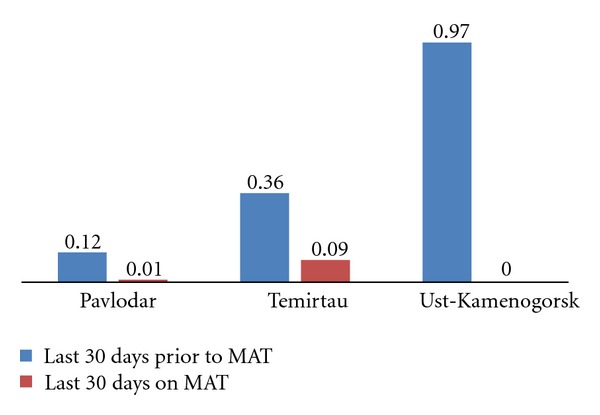
Use of opiates according to mean Opiate Treatment Index scores in patients before and after enrollment into medication assistance therapy, by site.

A significant difference in the frequency of opiate use by patients during the last 30 days prior to starting MAT and during the last 30 days on MAT was observed in Pavlodar and Ust-Kamenogorsk: mean frequency score of opiate use by patients in Pavlodar prior to MAT dropped from 0.12 (M = 0.12,  SD = 0.30) to 0.01 during the last 30 days on MAT (M = 0.01,  SD = 0.08); *t*(41) = 2.2,  *P* < 0.05) and in Ust-Kamenogorsk it dropped from 0.97 (M = 0.97,  SD = 1.9) to 0.00 (M = 0.00,  SD = 0.00); *t*(28) = 2.73,  *P* < 0.05). In Temirtau the frequency of opiate use also dropped, but this change was not statistically significant.  Figure 4 below demonstrates the mean differences in opiate use.

**Figure 5:**
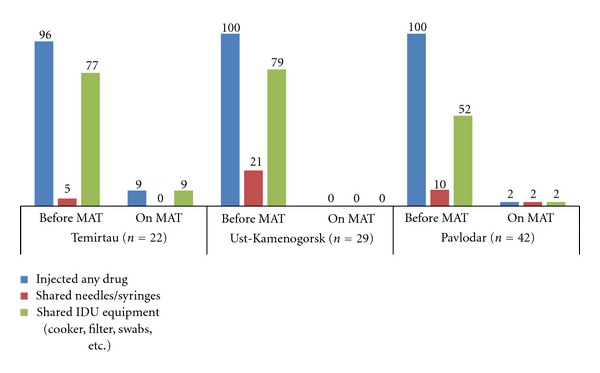
Drug injection practices, in %.

The assessment results showed that participation in MAT for at least three months resulted in a statistically significant reduction in HIV risk related to drug-taking behavior. The percentage of participants who injected any drug decreased from 95% during the last 30 days before enrolling in MAT to 9% during the last 30 days on MAT in Temirtau (*P* < 0.001); from 100% to 0% in Ust-Kamenogorsk (*P* < 0.0001); from 100% to 2% in Pavlodar (*P* < 0.001). Similarly, the proportion of persons who shared any injection equipment (cooker, filter, swabs, etc.) reduced from 77% to 9% in Temirtau (*P* < 0.001); from 79% to 0% in Ust-Kamenogorsk (*P* < 0.001); from 52% to 2% in Pavlodar (*P* < 0.0001). Reductions in sharing syringes and needles were seen across all three sites; however, this parameter was low at baseline and differences were not statistically significant: in Pavlodar, Temirtau, and Ust-Kamenogorsk 10%, 5%, and 21% of MAT patients reported sharing needles and syringes prior to entering MAT. During the last 30 days on MAT none of patients in Temirtau and Ust-Kamenogorsk and only 2% of patients in Pavlodar reported sharing syringes and needles (*P* > 0,05) (Figure 5). Low levels of syringes sharing at baseline can be explained by easy access to syringes via pharmacies and through network syringe exchange points that cover on average 47% of the estimated number PWID in Kazakhstan [19]. Similarly,no significant differences between low risk sexual behavior prior to MAT enrollment and on MAT were recorded.

**Figure 6:**
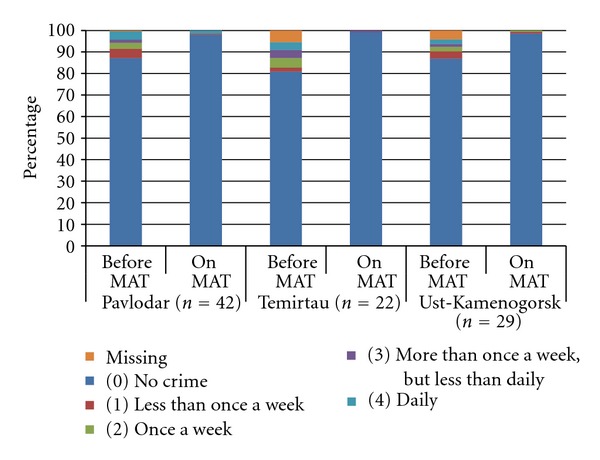
Criminal behavior before and after enrollment in medication-assisted therapy.

As shown in Figure 6, there were statistically significant reductions in engagement in criminal activity during the last 30 days by patients who participated in MAT for three months or longer compared to during the last 30 days prior to initiating MAT. As such, MAT patients in Pavlodar reported that 14% of them had committed some sort of crime (including fraud, drug dealing, sex work, violence, and/or property crime) before starting MAT and this figure was reduced to 2% after starting MAT (Wilcoxon signed ranks test: *Z* = −3.473,  *P* = 0.001). Similarly, initiation of MAT by patients in Ust-Kamenogorsk and Temirtau was associated with reductions in criminal behavior from 9% and 14% to 1% and 1%, respectively (*Z* = −3.025,  *P* = 0.002 and *Z* = −3.090,  *P* = 0.002). In addition, the data gathered suggests reductions in frequency of all types of criminal activities among patients compared to the period before MAT.

### 3.10. Drug Use Related Expenses

**Figure 7:**
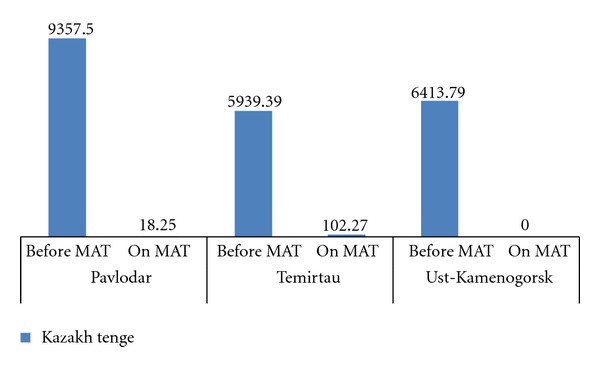
Mean daily expenditure in tenge for non-prescribed psychoactive substances by patients before and after enrollment in medication-assisted therapy, by site.

Patients were asked about their expenses for use of nonprescribed psychoactive substances on each of the last three days of use of those substances during MAT and just before starting MAT. As shown in Figure 7, patients' mean expenses for non-prescribed psychoactive substances on each single day of use prior to MAT were significantly greater than during MAT across all three sites: in Pavlodar (M = 9357.5 KZT ^2^, SD = 6184.7 KZ and M = 18.25 KZT, SD = 61.6 KZT); *t*(39) = 9.52,  *P* = 0.000); in Temirtau (M = 5939.4 KZT, SD = 4045.9 KZT and M = 102.3 KZT, SD = 288.9 KZT); *t*(21) = 6.94,  *P* = 0.000); in Ust-Kamenogorsk (M = 6413.8 KZT,  SD = 3905.5 KZT and M = 0.00 KZT, SD = 0.00 KZT); *t*(28) = 8.84,  *P* = 0.000). Respondents receiving MAT considered regained control over their own financial expenses and relief from a need to seek money for new doses of heroin as key benefits of MAT.

## 4. Limitations

One of the limitations of our study is that data collected on patients' criminal, drug use, and HIV risk behavior were based on self-report; however, self-reports about drug use during the last thirty days prior to the interview were well correlated with the results of urine toxicology tests performed during the same period, allowing us to regard self-reported behaviors as reliable. Also, evidence from researches that studied outcomes of methadone maintenance therapy in other countries [20–23] support the findings of our assessment.

## 5. Discussion and Conclusions

The GFTAM-funded pilot MAT project in Kazakhstan clearly demonstrates the feasibility and efficacy of prescription of methadone to treat opioid dependence in the local context. Between 46% and 61% of MAT patients at each pilot site were retained in the program at 12 months, which is similar to retention rates observed in other countries [24–26]. Of those retained in the program for 12 months or longer, between 41% and 92% of patients at each MAT site remained free from opioids. Patients reported that following the enrollment in MAT there was a decrease in their heroin use, risky drug injection behavior, spending on non-prescribed psychoactive substances, and criminal behavior, as well as an improvement in their health status. 

It is crucial to adopt evidence-based policies for the success of any health intervention, including MAT (6). The existing policies allow introducing MAT as a standard of care for treatment of opioid dependence in Kazakhstan. Furthermore methadone-based MAT may be provided in Kazakhstan at a relatively low cost: in 2011, the daily dose of methadone per patient was procured at US $1.00. This cost could be even lower if methadone was produced locally without dependence on external suppliers. 

Results of the assessment show several best practices that should be considered when scaling up MAT in Kazakhstan. In Pavlodar, MAT and other narcological services were effectively integrated with harm reduction programs, so that injection equipment and condoms could be accessed through a trust point located in the same building. The MAT site also supports the work of a patient self-help group, placing the nongovernmental organization's office adjacent to the MAT dispensing room. In addition, Pavlodar Narcology Center has arranged for an HIV specialist from the Oblast AIDS Center to work part time at the MAT site, to provide integrated HIV care for MAT patients which is in line with the best international practices and associated with decreased substance use, HIV, and health care utilization outcomes [26, 27]. 

The assessment also revealed some limitations that challenge effective implementation of MAT in Kazakhstan. Firstly, despite the legislature that enables provision of MAT and the highest political support, MAT program has been limited to only three cities of the country with a low number of patients enrolled. Biased attitudes towards MAT among the general public, medical professionals, and PWID, based upon incorrect information about the clinical and pharmacological features of opiate substitution therapy, have led active opposition to MAT. Some authors explain this opposition by the fact that Kazakhstan, as a former Soviet Union country, maintains close relationships with the Russian Federation and its older medical professionals are still much influenced by the Russian medical practice and theory [28]. Russia proactively promotes its well-known and internationally criticized antiopioid treatment policies [29, 30] employing various resources, including internet, mass media, professional medical journals, books, and conferences and these information channels still play a major role in the professional development of medical doctors in Central Asia. Secondly, training and technical assistance for MAT staff are currently provided as a part of the international development aid without the involvement of local medical education institutions and thus are not sustainable. An effective methadone procurement system is also lacking, and as a result there are often supply interruptions and the cost of the medication is unreasonably high.

The MAT monitoring and evaluation system is limited in that it is primarily focused on patient coverage and program expenditure indicators, with little attention paid to patient level outcomes or patient satisfaction. Clinicians providing MAT commonly rely on patients' feedback regarding the adequacy of their methadone dose as the sole measure of service quality. However our study found that while most patients gave high scores to the adequacy of their methadone dose to avoid experiencing withdrawal symptoms and craving drugs, they had a relatively modest perception of the quality of MAT services. This is an important observation as patients' satisfaction with services has been identified as a strong predictor of retention in treatment and better treatment outcomes [4, 31, 32].

Other limitations of the pilot MAT program include facility infrastructure and availability of services. There is a need for more patient-friendly locations for MAT sites, as well as adequate space for patient counseling. The opening hours of MAT sites are not always responsive to patients' needs, as patients are obliged to visit the narcological clinic on a daily basis while meeting their social responsibilities including employment and family-related functions. The unregistered status of methadone in Kazakhstan does not allow for take-home doses, and as a result MAT is often interrupted when patients must undergo inpatient treatment in other medical facilities or move away from their home cities. Such restrictive dispensing policy is maintained despite a growing body of evidence that take-home doses of methadone improve treatment outcomes and reduce health care costs [3, 33, 34]. In addition MAT is not available in the penitentiary system, which not only results in treatment interruption for incarcerated patients but also seriously limits the health care system's ability to control HIV and other blood-borne diseases among opioid dependent prison population. International evidence suggests that MAT in prison settings can be as equally beneficial as in community settings, helping opioid dependent inmates access health care services, increase adherence to ART when indicated, and reduce criminality and HIV risk behaviors [35, 36].

In order to prevent further expansion of the HIV epidemic, the government of Kazakhstan should support staged expansion of MAT starting with localities with a high prevalence of intravenous opioid use and HIV among PWID, followed by other places in the country where there is a need for such therapy. Such expansion should be implemented in accordance with the target coverage and quality indicators recommended by the WHO, UNAIDS, and UNODC [37]. Further expansion can be attained through training and authorization of narcologists at outpatient departments of dispensaries to prescribe MAT to opioid dependent patients in their catchment areas. Doing so would contribute to scaling up MAT availability and would also reduce the workload of narcologists currently working in the pilot MAT project, who currently are the only providers authorized to prescribe methadone.

The assessment results demonstrated insufficiency in MAT related specialist training that limits further expansion of this treatment method. To strengthen staff capacity building, updated information on MAT should be integrated into graduate and postgraduate medical curricula and qualified local professionals, including addiction psychiatry specialists, should be trained and engaged to work as technical advisors to support MAT sites in the provision of quality services consistent with national and international guidelines. 

Considering the wealth of knowledge gained during the MAT pilot phase, the existing clinical guidelines and standards on MAT should be revised based on lessons learned and WHO recommendations. This includes allowing the provision of MAT outside of narcological facilities, such as in correctional settings and nonnarcological hospitals; revision of admission and discharge criteria to ensure that the maximum number of PWID in need of MAT benefit from and are retained in treatment; and expanding the hours of operation at MAT sites. 

Given previous disruptions in methadone procurement and supply, the Kazakhstan's Ministry of Health should establish a centralized state-controlled mechanism of procurement and distribution of medications for MAT. Procurement should be properly planned considering all of the factors affecting time of actual product delivery, including tendering, licensing requirements, import procedures, and customs clearance. Regular monitoring of procurement performance should be established in order to address emerging challenges in a timely manner [38].

Improvements in monitoring and evaluation procedures should aim to ensure collection and analysis of data related not only to program implementation but also to its impact on patient behavior and health. It is also important to ensure standardization and simplification of data collection and reporting forms from various sites. Introduction of reliable health management information systems can increase data quality as well as clinical and programmatic decision making.

It is essential to develop comprehensive advocacy and communication strategies for MAT in order to deliver easy to comprehend evidence-based information for medical professionals and the general public, thus reducing the negative impact of false information. Nongovernment and community-based organizations should be engaged in such activities as intensively as possible, particularly to promote MAT among PWID and their families. Such organizations can include self-organized groups of MAT patients as in Pavlodar or parents of MAT patients such as in Ukraine [39].

Finally, we recommend that the Ministry of Health continues to make evidence-based decisions regarding the development of HIV and drug dependence treatment services and strengthens its emphasis on state-of-the-art research data, such as Cochrane reviews, that repeatedly confirm the safety and effectiveness of MAT compared to other methods of treatment for opiate addiction [40–42]. It is our pleasure to note that based on the findings and recommendations of our assessment report the Ministry of Health of the Republic of Kazakhstan has decided to expand MAT to two additional sites in the country [43, 44].

## Figures and Tables

**Table 1:** Characteristics of MAT patients who participated in interviews.

Site	Age Mean (SD)	Gender (men) Percentage	Completed years of school Mean (SD)	Months since enrollment Mean (SD)	Years of injecting drugs before MAT Mean (SD)
Pavlodar	34,7 (7,26)	69	9,3 (1,30)	17,1 (10,4)	12,4 (5,4)
Ust-Kamenogorsk	32,6 (4,56)	72	9,6 (2,03)	10,2 (4,3)	11,3 (4,9)
Temirtau	36,0 (7,55)	77	9,6 (1,19)	17,3 (12,9)	14,6 (4,6)

**Table 2:** Variables reported by medication-assisted treatment sites in Kazakhstan to the Republican Applied Research Center for Medicosocial Problems of Drug Addiction (RARC) and Republican AIDS Center (RAC).

Variables reported to the RARC	Variables reported to the RAC
(i) Patients' personal data	(i) Patients' sociodemographic profile
(ii) Patients' sociodemographic profile	(ii) Patients' biopsychosocial status
(iii) Patients' biopsychosocial status	(iii) Average daily dose of methadone per patient
(iv) Years of drug use	(iv) Remaining amount of methadone
(v) Information about types of treatment currently and previously received	(v) Number of new patients
(vi) Date of initiation of MAT	(vi) Number of dropouts and reasons for dropout
(vii) Clinical diagnosis based on ICD-10	(vii) Criminal charges
(viii) Daily dose of methadone prescribed	(viii) Concurrent illnesses including HIV, hepatitis B (HBV), hepatitis C (HCV), and tuberculosis
(ix) Changes in prescribed dose of methadone and reason for the changes	
(x) Number of new patients	
(xi) Number of dropouts and reasons for dropout	
(xii) Criminal charges	
(xiii) Concurrent illnesses including HIV, hepatitis B, hepatitis C, and tuberculosis	
(xiv) Laboratory test results	
(xv) Results of psychological assessment with dates	
(a) Short form of Minnesota Multiphasic Personality Inventory (MMPI-Short)	
(b) Addiction Severity Index	
(c) Zung Self-Rating Depression Scale	
(d) WHO QOL-100 (Quality of Life)	
(xvi) Description of side effects related to MAT with observation dates	
(xvii) Description of changes in patients' social well-being	
(xvii) Outcomes of therapy	
(xix) Reasons for exclusion from MAT (if applicable)	

**Table 3:** Results of medical chart review for patients on medication-assisted therapy, by treatment site.

Indicator	Pavlodar	Temirtau	Ust-Kamenogorsk
Proportion of patients on MAT with at least one complete clinical review in the last quarter	0	0	0
Proportion of MAT patients screened for hepatitis B	57%	85%	62%
Proportion of MAT patients screened for hepatitis C	77%	94%	59%
Proportion of patients on MAT with at least one psychosocial counseling session during the last month	21%	51%	94%
Proportion of patients who remained free from nonprescribed opioids^1^ six months after initiation of MAT	95%	68%	95%
Proportion of patients on MAT remaining in care six months after initiation of MAT	72%	55%	65%
Proportion of patients who remained free from nonprescribed opioids^1^ twelve months after initiation of MAT	92%	41%	84%
Proportion of patients on MAT remaining in care twelve months after initiation of MAT	61%	46%	61%
Proportion of patients on MAT with at least one sexual- and drug-related risk assessment completed during the last month	0%	0%	0%
Mean daily dose of methadone received by patients enrolled in MAT for three months or longer, mg (standard deviation)	66 (23,9)	69 (22,7)	73 (40,4)

^1^Based on urine toxicology test.

**Table 4:** Reasons for patient discharge from medication-assisted therapy in Kazakhstan, by treatment site.

	Pavlodar	Temirtau	Ust-Kamenogorsk
	*N* (%)	*N* (%)	*N* (%)
Total number of patients ever enrolled	102	85	78
Total number of patients discharged from MAT	54	50	43
Reasons for discharge from MAT			
Criminal charges	5 (4,9)	2 (2,4)	5 (6,4)
Personal life circumstances (voluntary)	18 (17,6)	11 (12,9)	16 (20,5)
Continued breach of MAT program rules	6 (5,9)	16 (18,8)	6 (7,7)
Completion of therapy (after methadone tapering)	20 (19,6)	5 (5,9)	6 (7,7)
Change of country of residence	2 (1,9)	3 (3,5)	9 (11,5)
Inpatient treatment	3 (2,9)	11 (12,9)	0
Death caused by concurrent illnesses	0	2 (2,4)	1 (1,3)

**Table 5:** Opiate treatment index scores interpretation table.

Frequency/quantity	Score
Abstinence	0.00
Once a week or less	0.01–0.13
More than once a week	0.14–0.99
Daily	1.00–1.99
More than once a day	2.00 or more

## Endnotes

1. Narcology is a subspecialty of psychiatry that deals with diagnosis and treatment of drug dependence. Narcologist is a medical doctor who specializes in narcology and provides narcological services.
2. KZT: Kazakh tenge. 1 USD = 146.35 KZT—exchange rate on February 16, 2012 obtained from http://www.nationalbank.kz.
